# Access to inpatient palliative care among cancer patients in France: an analysis based on the national cancer cohort

**DOI:** 10.1186/s12913-020-05667-8

**Published:** 2020-08-26

**Authors:** Asmaa Janah, Christine Le Bihan-Benjamin, Julien Mancini, Anne-Déborah Bouhnik, Philippe-Jean Bousquet, Marc-Karim Bendiane

**Affiliations:** 1 Aix Marseille Univ, INSERM, IRD, Economics and Social Sciences Applied to Health & Analysis of Medical Information (SESSTIM), 27 Boulevard Jean Moulin, Marseille, France; 2 Department of Health Data and Assessment, Survey Data Science and Assessment Division, French National Cancer Institute (Institut National du Cancer INCa), 52 Avenue André Morizet, Boulogne-Billancourt, France; 3 APHM, La Timone Hospital, BiosTIC, 264 Rue Saint-Pierre, Marseille, France; 4 Survey Data Science and Assessment Division, French National Cancer Institute (Institut National du Cancer INCa), 52 Avenue André Morizet, Boulogne-Billancourt, France

**Keywords:** Palliative care (PC), Access, Cancer diagnosis, French cancer cohort, National health system database

## Abstract

**Background:**

Closely linked to the concept of supportive care, the integrated model of palliative care (PC) implies identifying, assessing and treating physical and psychological suffering as early as needed, irrespective of patient characteristics. In France, as in the most southern European countries, little is known about the proportion of cancer patients who have access to PC. Accordingly, we aimed in this study to estimate the proportion of cancer patients in France who have access to inpatient PC, and to explore associated factors. We carried out a nationwide retrospective cohort study using data from the French national health system database (SNDS) for all individuals diagnosed with cancer in 2013 and followed between 2013 and 2016. We compared patients who had inpatient PC with those who did not.

**Results:**

Of the 313,059 cancer patients included in the national French cancer cohort in 2013, 53,437 (17%) accessed inpatient PC at least once between 2013 and 2016, ranging from 2% in survivors to 56% in the deceased population. Multivariate logistic regression revealed that women and younger patients (18–49 years old) were less likely to access inpatient PC while patients with a greater number of comorbidities, metastatic cancer, or cancer of the nervous system, were more likely to have done so.

**Conclusions:**

A negligible proportion of cancer survivors accessed inpatient PC. More research and training are needed to convince healthcare providers, patients, and families about the substantial benefits of PC, and to promote better integration of PC and oncology.

## Background

The increase in the number of people directly or indirectly affected by cancer is due to many factors, including increased incidence and improved survival [1]. In recent years, the international medical literature has shown that cancer patients, experience many deleterious and persistent effects which are either a direct result of their underlying disease, or a consequence of treatment [2–4]. These effects negatively influence both physical and psychological well-being. Adequate care is therefore needed which focuses not only on the disease, but also on patients and their families. Having the same objectives as supportive care in oncology, the integrated model of palliative care (PC) is based on the intervention of an interdisciplinary team which identifies, assesses and provides early treatment initiation for physical and psychological suffering in people with a life-threatening illness. Contrary to common perception, it is not limited to end-of-life situations [5, 6].

A great deal of attention is currently being focused on how and when to integrate PC into oncology [6–9]. Several studies have shown that an integrated model of PC throughout the care trajectory should remain close to early PC model and may provide benefits for both patients and health systems. Early PC has been reported to provide effective symptom management, enhance patients’ physical and mental health, and increase survival rates [10–12]. Indeed, the exponential increase in cancer treatments and the number of patients living longer with advanced disease has accentuated the recommendation to integrate PC at an early stage in the cancer trajectory, especially for patients with advanced cancer, or those who at the time of diagnosis have a high symptom burden due to psychological or spiritual distress [6]. However, PC is often introduced late in the course of the disease, especially when no therapeutic options remain and curative treatment is discontinued [13–15]. Several barriers to access to adequate PC have been reported, including clinicians’ lack of education about PC practices, their lack of confidence in their ability to effectively manage patients’ symptoms, and their insufficient knowledge of patients’ needs and the benefits which PC can bring [16–19].

Over the past fifteen years in France, decision-makers have made several attempts to improve cancer patient care. These include the implementation of cancer plans, pain management plans, PC and end-of-life care plans, as well as the adoption of the ‘Patient’s Rights and End-of-life Care’ Act clarifying end-of-life medical practices [20–25]. We provide through this study an overview of PC access in France between 2013 and 2016, and we evaluate the impact of the above measures, some already implemented in 2013 [21, 23–25] and others implemented during the 2013–2016 study period (e.g., the most recent national cancer plan (2014–2019) [20] and the most recent national PC plan (2015–2018, 22)). In France, all healthcare professionals are concerned by the palliative approach and are invited to integrate it into their practices. The family physician, specialist in general medicine, geriatrician, or other specialist, are the first concerned when this care is provided at home. At the hospital, the provided care is graduated to meet the patient’s needs according to the severity and the complexity of their health condition (i.e. acute care and rehabilitation units, PC beds and PC units).

While a large number of studies have explored PC access by cancer patients who subsequently die - including a previous study by our team on 2-year mortality [26] - studies on survivors who survive beyond 2 years after diagnosis and who access PC are very scarce. To tackle the lack of knowledge in this area, the main aim of this study was to estimate the proportion and describe the characteristics of cancer patients in France diagnosed in 2013 who benefited from inpatient PC at least once between 2013 and 2016, using a national exhaustive database. The secondary aim was to identify predictors of PC access in this population.

## Methods

### Study design

For this nationwide retrospective cohort study, we used data from the French National Cancer Cohort, which includes all people living in France with health insurance coverage (i.e., nearly 100% of the French population) diagnosed with and treated for cancer. A detailed description of the methodology can be found elsewhere [27].

### Data sources

The French National Cancer Cohort is extracted from the French national health system database (*Système National des Données de Santé, SNDS*), and the following data have been collected since 2010: 1) all individual healthcare utilization reimbursement data, collected in a single database from various national health insurance schemes (*Données de Consommation Inter-Régimes database, DCIR*); 2) private and public hospital database records, collected in the medical information system program database (*Programme de Médicalisation des Systèmes d’Information, PMSI*) by the national agency for information on hospital care. The PMSI database, which is based on diagnosis-related groups (DRG), describes hospital stays and costs in acute care units (*Médecine, Chirurgie, Obstétrique et Odontologie* - *MCO*), rehabilitation care units (*Soins de Suite et Réadaptation, SSR*), psychiatric units and hospital-at-home services (*Hospitalisation À Domicile*, *HAD*). The latter is a form of hospitalization that makes it possible to provide important medical and paramedical care at home, for a limited but renewable period depending on the evolution of the patient’s state of health. Specifically, the PMSI database contains demographic and medical information including diagnoses and medical procedures.

### Study population

In the present study, we included all cancer patients meeting the following inclusion criteria: 1) being entered as a new case in the national cohort in 2013, 2) insured under the National General Insurance scheme (i.e., nearly 90% of the French population) as of the end of 2013, 3) not having a tumor with uncertain or unknown behavior (i.e. whether malignant or benign), 4) receiving surgery, chemotherapy, hormone therapy, radiotherapy, inpatient PC or short-stay hospitalization for cancer for another reason in conventional medical units in 2013. Individuals who had a missing first inpatient or first outpatient treatment date were excluded from the analysis (i.e., dates of cancer related treatment before or after 2013).

### Outcomes

Access to inpatient PC was the primary outcome. Using the International Statistical Classification of Diseases and Related Health Problems - 10th Revision (ICD-10) “palliative care” coding, hospitalizations were identified according to whether they were linked to primary diagnosis, related diagnosis, or significant associated diagnosis. More specifically, inpatient PC stays were identified using: a) the ICD-10 PC code for each PC stay in acute care units or rehabilitation units (Z51.5), and b) the French code for support in hospital-at-home services (=04). Inpatient stays in PC beds in acute care units and admissions to inpatient PC units were also considered. Collected every year, the PMSI data are exhaustive and accessible. This made it possible, in particular using the ICD-10 codes, to estimate the number of patients with a PC code irrespective of the hospital setting (*MCO, HAD, SSR*), and therefore the number of patients who actually accessed PC, provided that the coding rules in the SNDS/PMSI have been fulfilled. As secondary outcomes, we calculated the following time intervals: time between initial inpatient PC access and death, cancer diagnosis and death, and diagnosis and first inpatient PC access.

### Individual characteristics

We extracted personal, socioeconomic, and medical data, including long-term disease (LTD) status. In France, patients with LTD status are entitled to 100% reimbursement for healthcare. Cancer is an LTD whose diagnosis is coded according to the ICD-10. Data on outpatient healthcare utilization and costs including retroceded drugs (i.e. drugs prescribed at the hospital but dispensed in community pharmacies) were also extracted. Cancer stages for included patients were identified using ICD-10 codes, since SNDS database does not provide clinical variables such as the TNM (Tumor, Nodes and Metastasis) cancer classification [27]. Furthermore, comorbidities were identified for included patients on the basis of the SNDS and the use of algorithms to distribute beneficiaries into 56 non-exclusive disease groups of chronic diseases, health events and chronic treatments, assembled into 13 main categories [28, 29] Finally, we used the French ecological deprivation index (Fdep99) as a proxy to consider patients’ socioeconomic status [30].

### Statistical analysis

Using the extracted data, we identified two groups: 1) individuals who accessed inpatient PC at least once since diagnosis, and 2) individuals who did not. Note that all individuals included in the study were followed for 3 years from diagnosis (i.e., 1095 days) unless death occurred.

First, we compared the distribution of socio-demographic and medical characteristics between these two groups. Second, independent factors associated with PC access were identified by performing multivariable logistic regressions while adjusting for inclusion characteristics. An initial model was fitted for the population followed between 2013 and 2016, only for the 11 investigated cancer sites common to both men and women (Table 1). Two additional models were fitted separately for survivors and the deceased population. Time intervals between the date of diagnosis and the date of access to the first inpatient PC (i.e., secondary outcomes) were subsequently analyzed using estimates of cumulative risk curves (Kaplan-Meier method), which enabled us to estimate the evolution in the cumulative probability of accessing PC. Finally, to explore the timing of PC in the disease trajectory, we computed the time intervals between diagnosis and PC access for the whole study population, and then according to vital status and several other patient characteristics (e.g., gender, age, etc.).

**Table 1:** Characteristics of cancer patients included in 2013 and followed between 2013 and 2016

	Total	Not accessing inpatient Palliative Care (2013–2016)	Accessing inpatient Palliative Care (2013–2016)
TOTAL (N, row %)	313,059 (100%)	259,622 (83%)	53,437 (17%)
Age in 2013 ^a^			
Age (Mean [SD])	64.4 [16.4]	63.4 [16.6]	69.4 [14.4]
Age (Median, [Q1-Q3])	66.0 [55.0–77.0]	65.0 [54.0–76.0]	70.0 [60.0–81.0]
Age in 2013			
Under 18 years	2967 (1%)	2727 (1%)	240 (< 1%)
18–49	50,285 (16%)	46,211 (18%)	4074 (8%)
50–74	167,369 (53%)	139,836 (54%)	27,533 (52%)
75 years and older	92,438 (30%)	70,848 (27%)	21,590 (40%)
Gender			
Male	153,019 (49%)	122,832 (47%)	30,187 (56%)
Female	160,040 (51%)	136,790 (53%)	23,250 (44%)
Cancer sites			
Gastro-intestinal^b^	53,064 (17%)	36,927 (14%)	16,137 (30%)
Respiratory^b^	22,850 (7%)	11,850 (5%)	11,000 (21%)
Endocrine glands^b^	6395 (2%)	6210 (2%)	185 (1%)
Hematologic^b^	20,394 (7%)	17,559 (7%)	2835 (5%)
Eye^b^	564 (< 1%)	526 (< 1%)	38 (< 1%)
Female genitals	16,757 (6%)	14,537 (6%)	2220 (4%)
Male genitals	28,177 (9%)	26,536 (10%)	1641 (3%)
Bone^b^	627 (< 1%)	517 (1%)	110 (1%)
Skin^b^	37,591 (12%)	36,227 (14%)	1364 (3%)
Breast	42,025 (13%)	39,693 (15%)	2332 (4%)
Nervous system^b^	4515 (1%)	2780 (1%)	1735 (3%)
Soft tissues^b^	906 (< 1%)	828 (< 1%)	78 (< 1%)
Upper aerodigestive tract^b^	9935 (3%)	7533 (3%)	2402 (4%)
Urinary tract^b^	19,198 (6%)	16,380 (6%)	2818 (5%)
Multiples sites	20,553 (7%)	13,316 (5%)	7237 (14%)
Non-attributable sites^%^	29,508 (10%)	28,203 (11%)	1305 (2%)
Cancer stage at diagnosis (2013)			
In situ	10,910 (4%)	10,763 (4%)	147 (1%)
Invasive	210,514 (67%)	184,628 (71%)	25,886 (48%)
Node involvement	17,322 (5%)	13,855 (5%)	3467 (6%)
Metastatic	39,767 (13%)	16,424 (6%)	23,343 (44%)
Non-attributable^%^	34,546 (11%)	33,952 (14%)	594 (1%)
Comorbidities in 2013			
No category	153,636 (49%)	136,615 (53%)	17,021 (32%)
1 category	91,963 (29%)	73,731 (28%)	18,232 (34%)
2 categories or more	67,460 (22%)	49,276 (19%)	18,184 (34%)
Death (2013–2016)			
No	226,695 (72%)	221,723 (85%)	4972 (9%)
Yes	86,364 (28%)	37,899 (15%)	48,465 (91%)
Area-level degree of social deprivation			
1-Very little deprivation (1st quintile)	60,350 (19%)	51,265 (20%)	9085 (17%)
2-Little deprivation (2nd quintile)	59,822 (19%)	50,190 (19%)	9632 (18%)
3-Moderate deprivation (3rd quintile)	60,829 (20%)	50,754 (20%)	10,075 (19%)
4-Substantial deprivation (4th quintile)	60,280 (19%)	49,542 (19%)	10,738 (20%)
5-Very high deprivation (5th quintile)	60,384 (19%)	48,274 (19%)	12,110 (23%)
Missing	11,394 (4%)	9597 (3%)	1797 (3%)
Region			
Auvergne-Rhône-Alpes	36,340 (12%)	29,728 (11%)	6612 (12%)
Bourgogne-Franche-Comté	13,192 (4%)	10,932 (4%)	2260 (4%)
Bretagne	14,363 (5%)	12,187 (5%)	2176 (4%)
Centre-Val de Loire	11,860 (4%)	9530 (4%)	2330 (4%)
Corse	1512 (< 1%)	1274 (< 1%)	238 (< 1%)
Départements d’Outre-Mer (DOM)/ Territoires d’Outre-Mer (TOM)	6474 (2%)	5420 (2%)	1054 (2%)
Grand Est	26,089 (8%)	21,240 (8%)	4849 (9%)
Hauts-de-France	28,035 (9%)	22,355 (9%)	5680 (11%)
Île-de-France (IDF)	53,663 (17%)	44,680 (17%)	8983 (17%)
Normandie	17,673 (6%)	14,617 (6%)	3056 (6%)
Nouvelle Aquitaine	26,536 (8%)	21,924 (8%)	4612 (9%)
Occitanie	25,966 (8%)	21,993 (8%)	3973 (7%)
Pays-de-la-Loire	17,695 (6%)	14,987 (6%)	2708 (5%)
Provence-Alpes-Côte d’Azur (PACA)	27,734 (9%)	23,662 (9%)	4072 (8%)
Unknown	5927 (2%)	5093 (3%)	834 (2%)

Table legend. ^a^ *SD* Standard Deviation, *Q1-Q3* Interquartile range, ^b^ Cancer sites common to both men and women (*N* = 11), ^%^ Cancer sites and stage which could not be characterized under the ICD-10 classification

The various sections of this article follow recommendations in the REporting studies Conducted using Observational Routinely-collected health Data (RECORD) statement [31].

## Results

### Proportion of patients accessing inpatient PC

**Fig. 1:**
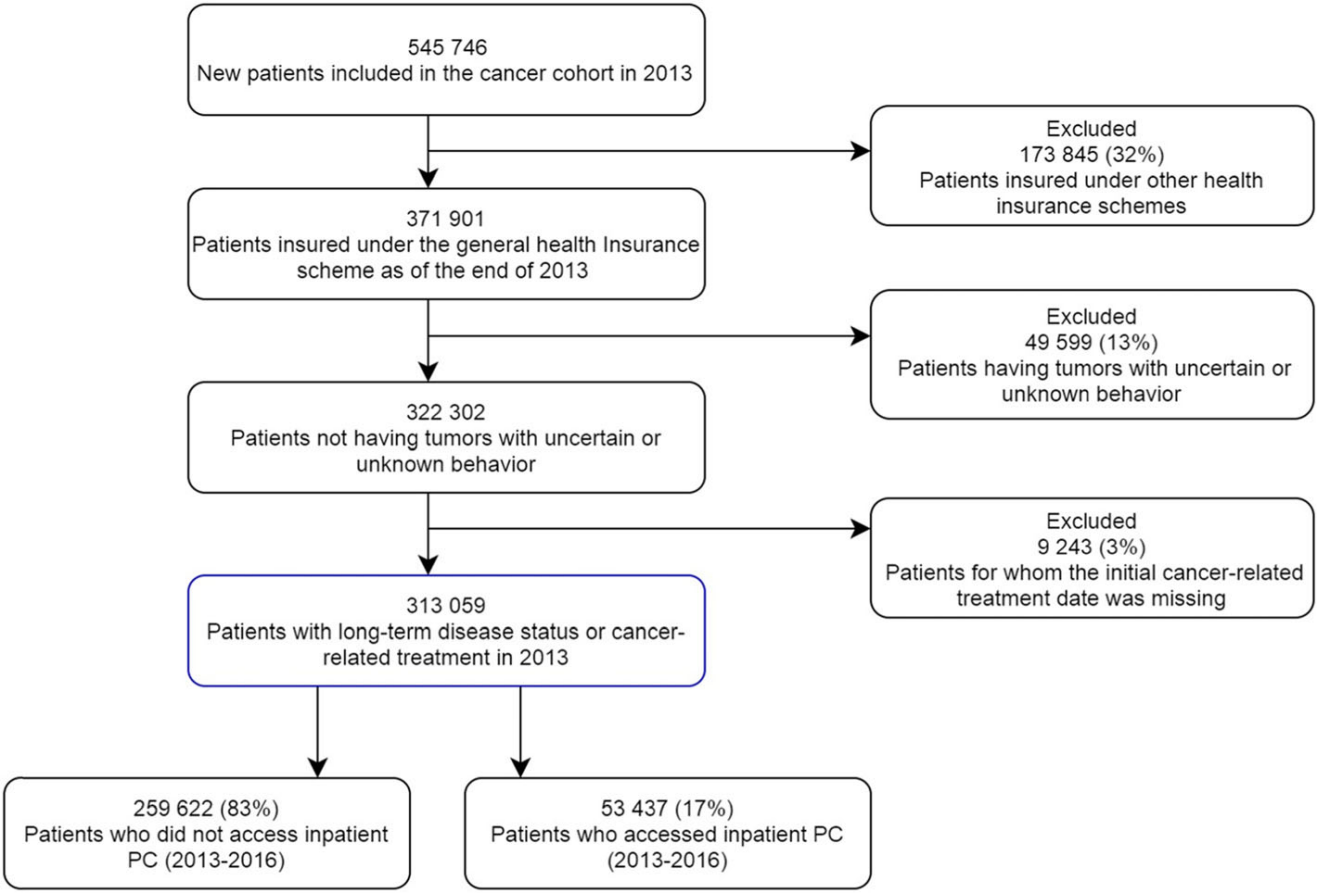
Selection of study population. PC: Palliative Care

Among the 313,059 patients included, 53,437 (17%) accessed inpatient PC at least once since diagnosis (Fig. 1): 88% during stays in acute care units, 17% in rehabilitation units and 15% in hospital-at-home services.

### Characteristics of patients who accessed PC at least once

The median age of patients who accessed PC at least once since diagnosis was 70 years. Fifty-two percent were aged 50–74 years in 2013 and 44% were women. As presented in Table 1, most patients who accessed PC had a cancer localized in the gastro-intestinal (30%) (*n* = 16,137) or respiratory (21%) (*n* = 11,000) systems. As regards to comorbidities, we observed that patients with no comorbidities were the less frequent to access inpatient PC (32%) (*n* = 17,021). Besides, most patients who accessed PC reside in the region Île-de-France (IDF) (17%) (*n* = 8983), while those residing in the region Corse had the less frequent inpatient PC access (< 1%) (*n* = 238). Note that among patients who accessed PC, the death rate reached 91% (*n* = 48,465). Among these, 10% died at home, 9% died in hospital-at-home services, 69% in acute care units and 12% in 17% in rehabilitation units.

Note that only 4972 (2%) of the patients still alive at the end of follow-up had accessed inpatient PC since their diagnosis. The number was much higher for patients who died between 2013 and 2016 (48,465 (56%) (Table 2). Note that the median age of patients who accessed PC at least once since diagnosis and who still alive at 3 years (i.e. *n* = 4972) was 66 years and 49% were women. Besides, most patients among these had a cancer localized in the gastro-intestinal (23%) (*n* = 1141), multiples sites (10%) (*n* = 520) or respiratory (10%) (*n* = 502).

**Table 2:** Characteristics of included cancer patients according to vital status

	Total	Death (2013–2016)	
		No	Yes
TOTAL (N, row %)	313,059 (100%)	226,695 (72%)	86,364 (28%)
Palliative Care access (2013–2016)			
No	259,622 (83%)	221,723 (98%)	37,899 (44%)
Yes	53,437 (17%)	4972 (2%)	48,465 (56%)
Cancer stage in 2013			
In situ	10,910 (4%)	10,432 (5%)	478 (1%)
Invasive	210,514 (67%)	164,098 (72%)	46,416 (54%)
Node involvement	17,322 (5%)	12,358 (5%)	4964 (6%)
Metastatic	39,767 (13%)	7426 (4%)	32,341 (37%)
Non-attributable^%^	34,546 (11%)	32,381 (14%)	2165 (2%)

Table legend. ^%^ Cancer stage which could not be characterized under the ICD-10 classification

During the 3 years of follow-up, 56% of people who accessed PC underwent inpatient chemotherapy, 48% surgery for cancer, and 38% had at least one session of radiotherapy (Supplementary file 1).

### Factors associated with PC access

Among patients with cancer in the eleven preselected sites common to both genders, women accessed inpatient PC significantly less often (adjusted odds ratio, aOR: 0.93; 95% Confidence Interval, 95% CI: 0.90–0.95), as did people aged 18–49 (aOR: 0.77; 95% CI: 0.73–0.81). Patients aged 75 years and older at diagnosis were significantly more likely to access inpatient PC. Compared with other cancer sites, patients with cancer of the nervous system were more likely to access inpatient PC access, while those with cancers of endocrine glands and skin were less likely. Accessing PC was also significantly higher in cancer patients with a metastatic cancer, those with a high number of comorbidities, and those living in a severely socially deprived area. In addition, patients residing in the three regions “Île-de-France”, “Hauts-de-France” and “Auvergne-Rhône-Alpes” had the most frequent PC access. Note that after stratification for vital status (i.e., still alive or deceased at 3 years), we found discordant results regarding age, gender, and comorbidities as predictors of accessing PC. More specifically, the stratified model focusing solely on deceased cancer patients showed that women, younger patients and those with fewer comorbidities were the most likely populations to access inpatient PC, while the results for the stratified model limited to survivors were comparable with those from the main model which took into account the entire incident population (i.e., patients aged 18–49 at diagnosis and those without comorbidities were the populations least likely to have accessed PC at least once). However, the impact of gender was not confirmed (Table 3). Note that additional analyses were carried out for the entire population (i.e. survivors and died patients) while including gender specific diagnoses and underlined the same associated factors with inpatient PC access than the initial analysis presented in the “Total” model in Table 3 (Supplementary file 2).

**Table 3:** Factors independently associated with Palliative Care access

	Adjusted odds ratios [95% confidence interval]		
	Accessed inpatient palliative care vs did not access (2013–2016)		
	Total (*N* = 170,599)	Death (2013–2016)	
		No (*N* = 109,793)	Yes (*N* = 60,806)
Gender (ref. male)			
Female	0.93 [0.90–0.95]	0.97 [0.90–1.05]	1.13 [1.09–1.17]
Age in 2013 (ref. 50–74)			
Under 18 years	0.44 [0.37–0.52]	0.92 [0.67–1.26]	1.77 [1.22–2.56]
18–49	0.77 [0.73–0.81]	0.88 [0.78–0.99]	1.39 [1.28–1.51]
75 years and older	1.61 [1.56–1.66]	1.34 [1.22–1.46]	0.82 [0.79–0.85]
Cancer site (ref. Respiratory track)			
Gastro-intestinal	0.66 [0.64–0.68]	0.64 [0.57–0.72]	0.99 [0.95–1.04]
Endocrine glands	0.08 [0.07–0.09]	0.14 [0.10–0.19]	0.57 [0.45–0.73]
Hematologic	0.39 [0.37–0.41]	0.66 [0.57–0.76]	0.62 [0.58–0.66]
Bone	0.48 [0.38–0.60]	0.62 [0.37–1.04]	0.97 [0.68–1.38]
Skin	0.09 [0.09–0.10]	0.11 [0.09–0.13]	0.34 [0.32–0.37]
Nervous system	1.61 [1.50–1.74]	0.63 [0.48–0.81]	1.76 [1.59–1.95]
Soft tissues	0.23 [0.18–0.29]	0.18 [0.08–0.37]	0.55 [0.40–0.76]
Upper aerodigestive tract	0.66 [0.63–0.70]	0.89 [0.77–1.04]	0.80 [0.74–0.86]
Urinary tract	0.33 [0.31–0.34]	0.33 [0.28–0.39]	0.82 [0.77–0.88]
Eye	0.21 [0.15–0.30]	0.21 [0.09–0.51]	0.75 [0.45–1.24]
Cancer stage (ref. Invasive)			
In situ	0.14 [0.12–0.17]	0.33 [0.23–0.47]	0.39 [0.30–0.51]
Node involvement	1.69 [1.60–1.78]	1.77 [1.55–2.03]	1.35 [1.26–1.46]
Metastatic	5.37 [5.19–5.55]	3.56 [3.17–4.00]	1.96 [1.89–2.04]
Non-attributable^a^	0.08 [0.06–0.10]	0.14 [0.08–0.22]	0.25 [0.17–0.35]
Comorbidities in 2013 (ref. no comorbidities)			
1 category	1.20 [1.17–1.24]	1.22 [1.12–1.33]	0.79 [0.75–0.82]
2 categories or more	1.37 [1.32–1.41]	1.58 [1.44–1.74]	0.66 [0.63–0.68]
Social deprivation level (ref. Very high deprivation (5th quintile))			
Very little deprivation (1st quintile)	0.86 [0.82–0.90]	0.74 [0.65–0.84]	1.06 [0.99–1.12]
Little deprivation (2nd quintile)	0.93 [0.89–0.97]	0.87 [0.77–0.98]	1.09 [1.03–1.15]
Moderate deprivation (3rd quintile)	0.92 [0.88–0.96]	0.96 [0.85–1.08]	1.02 [0.97–1.08]
Substantial deprivation (4th quintile)	0.94 [0.90–0.98]	0.87 [0.78–0.98]	1.03 [0.97–1.08]

Table legend. Multivariable logistic regressions were selected by a forward stepwise selection procedure (probability threshold = 20%, probability of staying in the model = 5%) and adjusted for region of residence. Only cancer sites common to both men and women were considered, ^a^ Cancer sites and stage which could not be characterized under the ICD-10 classification

### Cumulative probability of accessing inpatient PC since diagnosis

**Fig. 2:**
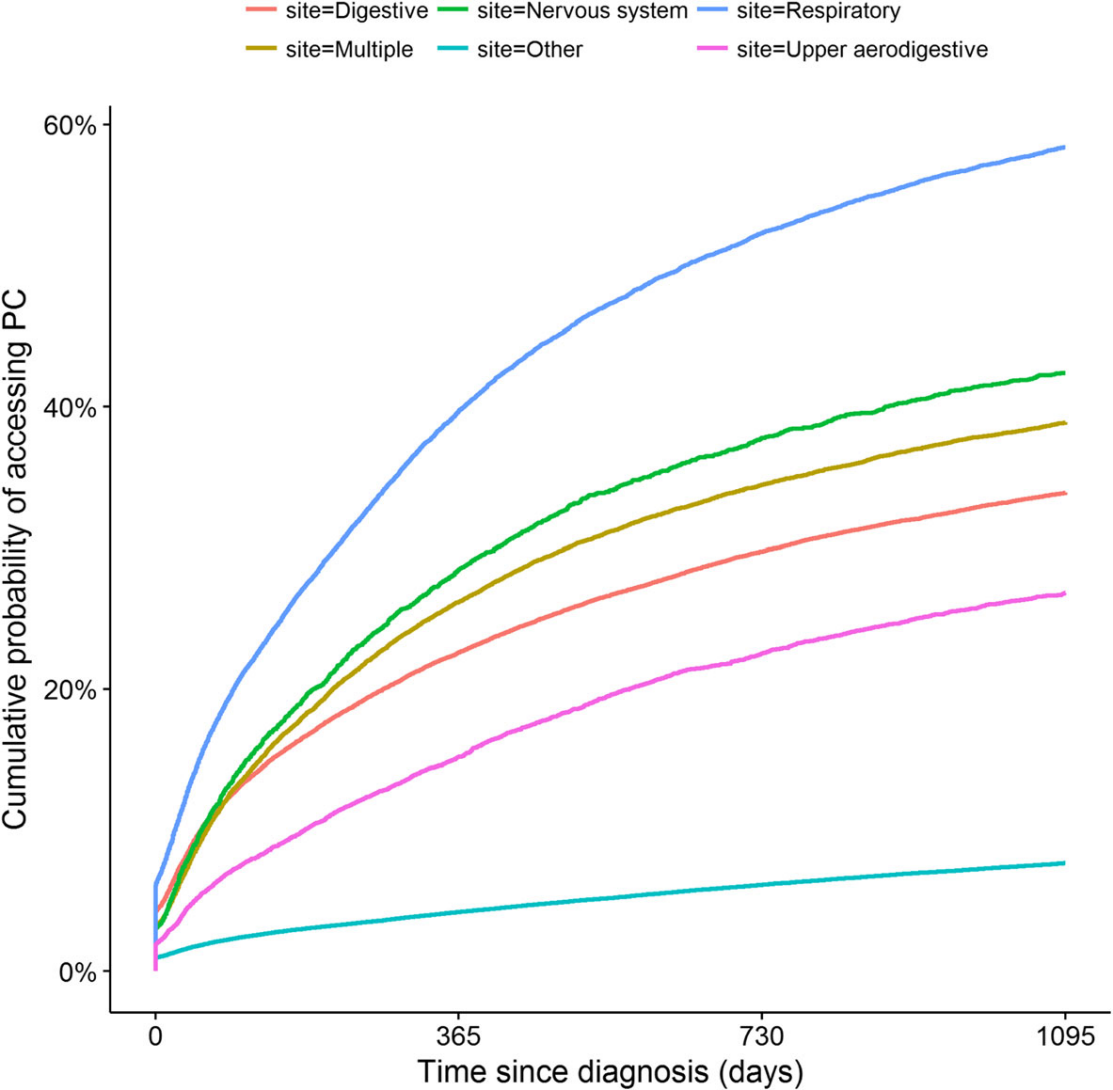
Kaplan–Meier curves of the cumulative probability of accessing Palliative Care according to cancer site. PC: Palliative Care, Site: Cancer site

Figure 2 & supplementary files 3, 4, 5, 6 and 7 present the cumulative probabilities of inpatient PC access according to cancer site, stratified for individual and medical characteristics. Overall, the Kaplan Meier survival curves showed that the probability of accessing PC, irrespective of gender, age, cancer stage, comorbidities and deprivation index, increased over time and tended to plateau at the end of the 3 years of follow-up. For example, the cumulative probability of accessing PC in patients with cancer of the respiratory system at 1, 2 and 3 years was, respectively, 40, 50 and 57% (Fig. 2). Note that these patients had the highest cumulative probabilities of accessing PC since diagnosis irrespective of age (Supplementary file 3), gender (Supplementary file 4), cancer stage (Supplementary file 5), comorbidities (Supplementary file 6) and social deprivation level (Supplementary file 7).

### Timing of PC access

Median time between diagnosis and initial PC access was 197 days. This interval was 204 and 187 days in men and women, respectively, and decreased with age. The median time intervals between diagnosis and PC access were 274, 283, 237, and 129 days for individuals aged under 18, 18–49, 50–74, and 75 years and older, respectively. When comparing patients who died with survivors at 3 years’ follow-up, the latter accessed inpatient PC earlier than the former. The median time intervals between diagnosis and PC access were 199 and 161 days, respectively, for deceased patients and survivors. Concerning the time between inpatient PC access and death, we noted an interval of 30 days. This median time interval was 29 and 33 days in men and women, respectively, and decreased as well with age.

## Discussion

### Main results

This is the first nationwide retrospective cohort study to provide a quantitative description of inpatient PC access in cancer patients in France, irrespective of vital status (i.e., alive or deceased), 3 years after diagnosis. The results revealed that less than one fifth of patients accessed inpatient PC after diagnosis, and that patients who died during follow-up were much more likely to access it. Furthermore, we found that men, older patients, those with cancer of the nervous system, people with metastatic cancer, and patients with one or more comorbidity were the most likely groups to access inpatient PC. Differences in PC access in terms of social deprivation and regions were also observed.

### Inpatient PC access since diagnosis

Seventeen percent of our study population accessed PC at least once since diagnosis, mostly during a stay in an acute care unit. Unlike several previous studies which investigated those who died during follow-up, the innovative element of this work is that it included individuals who were still alive three after diagnosis [13, 26, 32–34]. We found that 56% of those who died accessed inpatient PC, which is very similar to the 57% we found in a previous study exclusively exploring PC access for cancer patients who died between 2013 and 2015 [26]. This would suggest that at least among the deceased population, the prevalence of access to inpatient PC remains stable over time.

The very low overall rate of PC access in the present study (17%) is related to the fact that very few cancer survivors (2%) accessed it. One might consider that providing PC to cancer survivors is a waste of resources. However, we believe that all cancer patients, including survivors with a good or unknown survivorship prognosis, should be able to benefit from it. In a recent commission from The Lancet Oncology, Stein Kaasa and colleagues stated that the overall goal of PC is to improve patients’ quality of life congruent with their preferences from a patient-centered perspective. Accordingly, the commission not only challenges the conventional and dualistic perspective of dealing with the tumor or the patient by underlining PC needs but promotes a combined approach that places the patient’s perspective at the center of the care. They added that optimizing symptom management, and active involvement of patients and their families throughout the care trajectory can be achieved by integrating oncology and patient-tailored PC [6].

The misconceptions that healthcare providers, patients, and families may have about PC may be one of the principal reasons why patients tend to access it primarily near death [19, 35]. Many still do not understand that PC can be introduced alongside curative strategies irrespective of age or cancer stage. Indeed, in France, the notion that PC is only associated with end-of-life care is rooted in the PC movement. For example, in recent decades, PC has been raised in many debates and discussions surrounding physician-assisted suicide and active euthanasia (which are prohibited under French law). This has only served to strengthen the perception that PC is exclusively for end-of-life purposes [36, 37]. In a recent French qualitative study by Sarradon et al., the authors showed that once a medical team decide to refer a patient to PC services (especially when an advanced cancer stage is diagnosed), barriers to actually accessing PC increased, specifically because the term ‘palliative care’ is so closely associated with death and the terminally ill. Indeed, the authors found that doctors would be more willing to prescribe PC if the term could be avoided [36]. In France, the ongoing EPIC multicenter randomized clinical trial was launched in 2017 [38]. Aiming primarily to assess the benefits of early PC access in patients with upper gastrointestinal tract cancers, with a secondary aim of comparing results with those reported in recent highly regarded studies [10–12], EPIC could provide encouraging findings to promote the early-integration model of PC in French hospitals.

With regard to the location where PC is provided, we found that hospital-at-home services was the least frequent setting of those examined. We remind the reader that PC access occurring in outpatient settings was not considered in the present study. It should be noted that most PC in France is provided in hospitals, thanks to several national plans [22, 39, 40]. PC is very limited in home (NB: not under the hospital-at-home framework) and other outpatient contexts. Developing outpatient PC care is important, especially when promoting early integrated PC in oncologic care after diagnosis. Oncologic care is mainly delivered in the outpatient setting and is an ideal setting to comprehensively manage cancer patients’ physical and psychological symptoms [41, 42] .

There is increasing evidence to suggest that PC access depends on patients’ sociodemographic and medical characteristics [13, 14, 32, 43–47]. The results from the present study support this. For example, we found that men, older patients, and those with one or more comorbidities were the most likely subpopulations to access this care. However, these results are not consistent with those reported in our previous study which was limited to deceased cancer patients [26], or with those from other studies examining deceased patients [13, 32, 45, 46, 48]. This would suggest divergences related to the needs and preferences expressed by each study population (i.e., survivors or deceased patients) regarding PC, and in particular to what extent they are close to dying or not.

In addition, this study has shown some differences in access to PC in terms of social deprivation and regions. In fact, from 2000, consecutive five-year national public health plans including the creation of specific funding mechanisms were put in place in France not only to promote the development of PC but also to guarantee geographic continuity of care in all French territories, particularly in rural areas and overseas districts [20, 22, 25, 39]. In addition, like most healthcare in France, the PC is free for all patients who need it. The problem is that the provision of PC care is highly dependent on medical practices, the perception of medical staff of end-of-life patients and patient-doctor communication, as previously reported [37]. In addition, certain personal, social, and economic characteristics of included patients could not be considered in this study due to the type of data declared in the SNDS. However, data concerning the difference in access to inpatient PC regarding personal or social characteristics, except those considered in our analyzes, as age and gender and especially among cancer survivors, are very scarce. New specific studies are needed to explore the personal factors that may be associated with the perception of patients or medical staff regarding PC. Previous results of national surveys considering personal and psychological dimensions such as anxiety and religiosity, suggested that these could explain the possible reluctance to PC and then constitute barriers to access to specialized inpatient PC [37, 49, 50].

### Strengths and limitations

This is the largest cohort study conducted in France to explore inpatient PC access and its predictors, within 3 years of diagnosis and irrespective of vital status. Recognized as one of the largest cancer databases in the world, the national French Cancer Cohort allowed us to include a representative and comprehensive study population, in that it included varied cancer sites and stages, as well as patients still alive 3 years after diagnosis (and not only deceased patients). This follow-up period was chosen to reflect the acute phase after cancer diagnosis, with a view to evaluating the care provided during this phase, and not the care provided following possible recurrence and/or long-term complications.

Although the French national health system database (SNDS) allowed us to measure the overall delivered care, including inpatient PC, during the 3 years of follow-up, this data source has several limitations. First, it does not provide information on the primary cause of death for those who died between 2013 and 2016. Current national projects are working on chaining the causes of death in the SNDS. This will help to overcome this limitation in the future. Furthermore, the place of death will also become available. Second, apart from hospital-at-home stays, the SNDS databases does not contain information on care provided at home or in any other outpatient PC setting, (e.g., care delivered in old age homes, care delivered after an intervention by a mobile PC team). Accordingly, our study may underestimate PC access in this population. Third, we used the PMSI database to track inpatient PC access that occurred during 3 years of follow-up. However, like any database based on professionals inputting data on the care they provide, the PMSI only allows us to see whether PC was provided or not. The data does not allow us to judge the quality or the effect of the care provided. This limitation may become more problematic given that encoding defines the overall activity of the hospital and consequently its budget. This may lead to a possible overestimation of PC access in the present study.

## Conclusions

We found that less than one in five patients accessed inpatient PC since cancer diagnosis, most of the time in the period before death. Only 2% of cancer patients still alive 3 years after diagnosis had accessed inpatient PC. These two findings suggest the need for better and earlier integration of PC into oncology practices, and the need to consider the individual needs and preferences of cancer patients irrespective of cancer stage. Further research and education are urgently needed especially to overcome healthcare providers’, patients’ and families’ misguided belief that PC is exclusively for end-of-life care contexts.

## Supplementary information

**Additional file 1.** Treatments delivered since cancer diagnosis (2013–2016).

**Additional file 2.** Factors independently associated with Palliative Care access.

**Additional file 3.** Kaplan–Meier curves of the cumulative probability of accessing PC by age.

**Additional file 4.** Kaplan–Meier curves of the cumulative probability of accessing PC site by gender.

**Additional file 5.** Kaplan–Meier curves of the cumulative probability of accessing PC by cancer stage.

**Additional file 6.** Kaplan–Meier curves of the cumulative probability of accessing PC by comorbidity.

**Additional file 7.** Kaplan–Meier curves of the cumulative probability of PC access by deprivation level.

## Data Availability

Data are available from the French national cancer institute. For correspondence: Philippe Jean Bousquet; pjbousquet@institutcancer.fr

## Abbreviations

PC: Palliative Care
SNDS: Système National des Données de Santé
DCIR: Données de Consommation Inter-Régimes
PMSI: Programme de Médicalisation des Systèmes d’Information
DRG: Diagnosis-Related Groups
MCO: Médecine, Chirurgie, Obstétrique et Odontologie
SSR: Soins de Suite et Réadaptation
HAD: Hospitalisation À Domicile
ICD: International Statistical Classification of Diseases and Related Health Problems
LTD: Long-Term Disease
TNM: Tumor, Nodes and Metastasis
IDF: Île-de-France
DOM: Départements d’Outre-Mer / TOM: Territoires d’Outre-Mer
PACA: Provence-Alpes-Côte d’Azur
aOR: Adjusted Odds Ratio
95% CI: 95% confidence Interval
