# Supplementary material for: Access to inpatient palliative care among cancer patients in France: an analysis based on the national cancer cohort
Source: BMC Health Serv Res. 2020 Aug 26;20:798. doi: 10.1186/s12913-020-05667-8 (PMC7448507; doi:10.1186/s12913-020-05667-8)
Supplement: Supplementary file 1 — Additional file 1. Treatments delivered since cancer diagnosis (2013–2016). [file 12913_2020_5667_MOESM1_ESM.docx]

# Supplementary file 1. Treatments delivered since cancer diagnosis (2013-2016)

Figure legend. Among patients who accessed inpatient Palliative Care between 2013 and 2016, 48% underwent surgery for cancer
