# Supplementary material for: Access to inpatient palliative care among cancer patients in France: an analysis based on the national cancer cohort
Source: BMC Health Serv Res. 2020 Aug 26;20:798. doi: 10.1186/s12913-020-05667-8 (PMC7448507; doi:10.1186/s12913-020-05667-8)
Supplement: Supplementary file 2 — Additional file 2. Factors independently associated with Palliative Care access. [file 12913_2020_5667_MOESM2_ESM.docx]

# Supplementary file 2. Factors independently associated with Palliative Care access

|  | Adjusted Odds Ratios [95% Confidence Interval] | | |
| --- | --- | --- | --- |
|  | Access to inpatient palliative care vs. no access | | |
|  | Total  (N=313 059) | Men  (N= 153 019) | Women  (N= 160 040) |
| Gender (ref. Men) | | | |
| Women | 0.91 [0.88-0.93] | - | - |
| Age in 2013 (ref. 50-74 years) | | | |
| Less than 18 years | 0.43 [0.37-050] | 0.37 [0.30-0.46] | 0.50 [0.40-0.63] |
| 18-49 years | 0.75 [0.72-0.78] | 0.80 [0.75-0.84] | 0.74 [0.70-0.78] |
| 75 years and older | 1.73 [1.68-1.77] | 1.53 [1.48-1.58] | 1.99 [1.92-2.07] |
| Cancer site (ref. Respiratory system) | | | |
| Gastro-intestinal | 0.67 [0.64-0.69] | 0.69 [0.66-0.72] | 0.60 [0.57-0.64] |
| Endocrine glands | 0.08 [0.07-0.10] | 0.10 [0.08-0.13] | 0.07 [0.06-0.09] |
| Hematologic | 0.41 [0.39-0.44] | 0.41 [0.38-0.43] | 0.40 [0.37-0.43] |
| Eye | 0.23 [0.16-0.32] | 0.27 [0.17-0.42] | 0.17 [0.10-0.30] |
| Female genitals | 0.42 [0.39-0.44] | - | 0.39 [0.36-0.42] |
| Male genitals | 0.14 [0.13-0.15] | 0.14 [0.13-0.15] | - |
| Bone | 0.51 [0.40-0.64] | 0.48 [0.35-0.65] | 0.52 [0.37-0.72] |
| Skin | 0.10 [0.09-0.12] | 0.13 [0.14-0.13] | 0.08 [0.07-0.08] |
| Breast | 0.15 [0.14-0.15] | 0.14 [0.09-0.21] | 0.14 [0.13-0.15] |
| Nervous system | 1.75 [1.63-1.88] | 2.03 [1.84-2.24] | 1.43 [1.28-1.59] |
| Soft tissues | 0.24 [0.19-0.31] | 0.19 [0.13-0.27] | 0.30 [0.21-0.41] |
| Upper aerodigestive tract | 0.70 [0.66-0.75] | 0.70 [0.65-0.74] | 0.66 [0.59-0.75] |
| Urinary tract | 0.34 [0.32-0.36] | 0.32 [0.30-0.34] | 0.39 [0.36-0.43] |
| Multiples sites | 0.69 [0.66-0.72] | 0.74 [0.70-0.78] | 0.60 [0.56-0.65] |
| Non-attributable sites | 0.26 [0.24-0.28] | 0.28 [0.25-0.31] | 0.22 [0.19-0.24] |
| Cancer stage (ref. Invasive) | | | |
| In situ | 0.12 [0.10-0.14] | 0.16 [0.13-0.20] | 0.10 [0.08-0.12] |
| Node involvement | 1.77 [1.70-1.85] | 1.79 [1.69-1.91] | 1.76 [1.65-1.87] |
| Metastatic | 6.26 [6.09-6.43] | 5.58 [5.38-5.79] | 7.17 [6.88-7.47] |
| Non-attributable | 0.19 [0.17-0.21] | 0.20 [0.18-0.23] | 0.19 [0.16-0.22] |
| Comorbidities in 2013 (ref. No comorbidities) | | | |
| 1 | 1.24 [1.20-1.27] | 1.20 [1.16-1.25] | 1.27 [1.22-1.32] |
| >=2 | 1.43 [1.39-1.48] | 1.41 [1.35-1.46] | 1.45 [1.39-1.52] |
| Social deprivation level (ref. Very high deprivation (5th quintile)) | | | |
| Very little deprivation (1st quintile) | 0.83 [0.80-0.86] | 0.84 [0.80-0.88] | 0.83 [0.78-0.87] |
| Little deprivation (2nd quintile) | 0.91 [0.87-0.94] | 0.92 [0.88-0.96] | 0.89 [0.84-0.94] |
| Moderate deprivation (3rd quintile) | 0.90 [0.87-0.94] | 0.89 [0.85-0.94] | 0.92 [0.87-0.97] |
| Substantial deprivation (4th quintile) | 0.92 [0.89-0.96] | 0.93 [0.89-0.97] | 0.92 [0.87-0.97] |
| Regions (ref. Île-de-France (IDF)) | | | |
| Auvergne-Rhône-Alpes | 1.09 [1.05-1.14] | 1.09 [1.03-1.16] | 1.10 [1.03-1.17] |
| Bourgogne-Franche-Comté | 0.81 [0.76-0.86] | 0.75 [0.69-0.82] | 0.88 [0.81-0.97] |
| Bretagne | 0.83 [0.78-0.88] | 0.81 [0.75-0.88] | 0.85 [0.78-0.93] |
| Centre-Val de Loire | 1.08 [1.02-1.15] | 1.09 [1.01-1.19] | 1.06 [0.97-1.17] |
| Corse | 0.47 [0.20-1.10] | 0.58 [0.18-1.90] | 0.38 [0.11-1.33] |
| Départements d'Outre-Mer (DOM)/ Territoires d'Outre-Mer (TOM) | 0.80 [0.51-1.26] | 0.37 [0.16-0.86] | 1.30 [0.75-2.25] |
| Grand Est | 0.93 [0.88-0.97] | 0.90 [0.85-0.96] | 0.96 [0.89-1.03] |
| Hauts-de-France | 1.11 [1.06-1.16] | 1.09 [1.02-1.16] | 1.12 [1.04-1.20] |
| Normandie | 0.96 [0.91-1.02] | 0.95 [0.88-1.02] | 0.98 [0.90-1.07] |
| Nouvelle Aquitaine | 0.91 [0.87-0.95] | 0.90 [0.85-0.96] | 0.91 [0.85-0.98] |
| Occitanie | 0.82 [0.78-0.86] | 0.82 [0.77-0.88] | 0.82 [0.76-0.88] |
| Pays-de-la-Loire | 0.88 [0.83-0.93] | 0.86 [0.80-0.93] | 0.91 [0.83-0.99] |
| Provence-Alpes-Côte d'Azur (PACA) | 0.81 [0.77-0.85] | 0.83 [0.78-0.88] | 0.79[0.73-0.85] |
| Unknown | 0.89 [0.81-0.98] | 0.91 [0.81-1.03] | 0.83 [0.71-0.97] |

Table legend. Multivariable logistic regressions were selected by a forward stepwise selection procedure (probability threshold=20%, probability of staying in the model=5%). The entire population (i.e. survivors and died patients) was considered while including gender specific diagnoses.
