# Supplementary material for: Access to inpatient palliative care among cancer patients in France: an analysis based on the national cancer cohort
Source: BMC Health Serv Res. 2020 Aug 26;20:798. doi: 10.1186/s12913-020-05667-8 (PMC7448507; doi:10.1186/s12913-020-05667-8)
Supplement: Supplementary file 3 — Additional file 3. Kaplan–Meier curves of the cumulative probability of accessing PC by age. [file 12913_2020_5667_MOESM3_ESM.docx]

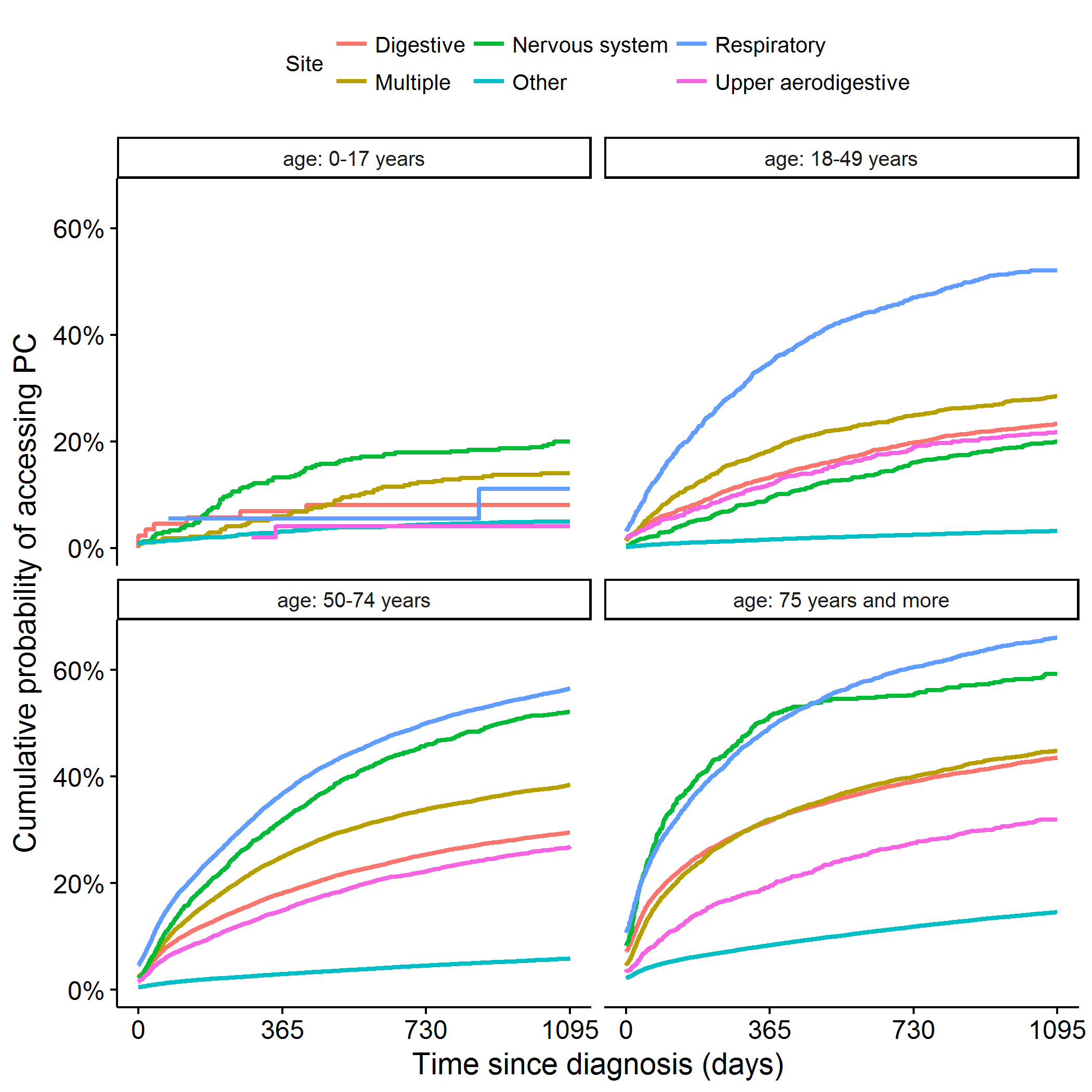


# Supplementary file 3. Kaplan–Meier curves of the cumulative probability of accessing PC by age

Figure legend. PC: Palliative Care, Site: Cancer site
